# Survival of pediatric patients after cardiopulmonary resuscitation for in-hospital cardiac arrest: a systematic review and meta-analysis

**DOI:** 10.1186/s13052-021-01058-9

**Published:** 2021-05-29

**Authors:** Melaku Bimerew, Adam Wondmieneh, Getnet Gedefaw, Teshome Gebremeskel, Asmamaw Demis, Addisu Getie

**Affiliations:** 1 Department of Nursing, College of Health Sciences, Woldia University, Woldia, Ethiopia; 2 Department of Midwifery, College of Health Sciences, Woldia University, Woldia, Ethiopia

**Keywords:** Cardiopulmonary resuscitation, Pediatric, Survival to hospital discharge

## Abstract

**Background:**

In-hospital cardiac arrest is a major public health issue. It is a serious condition; most probably end up with death within a few minutes even with corrective measures. However, cardiopulmonary resuscitation is expected to increase the probability of survival and prevent neurological disabilities in patients with cardiac arrest. Having a pooled prevalence of survival to hospital discharge after cardiopulmonary resuscitation is vital to develop strategies targeted to increase probability of survival among patients with cardiac arrest. Therefore, this systematic review and meta-analysis was aimed to assess the pooled prevalence of survival to hospital discharge among pediatric patients who underwent cardiopulmonary resuscitation for in-hospital cardiac arrest.

**Methods:**

PubMed, Google Scholar, and Cochrane review databases were searched. To have current (five-year) evidence, only studies published in 2016 to 2020 were included. The weighted inverse variance random-effects model at 95%CI was used to estimate the pooled prevalence of survival. Heterogeneity assessment, test of publication bias, and subgroup analyses were also employed accordingly.

**Results:**

Twenty-five articles with a total sample size of 28,479 children were included in the final analysis. The pooled prevalence of survival to hospital discharge was found to be 46% (95% CI = 43.0–50.0%; I^2^ = 96.7%; *p* < 0.001). Based on subgroup analysis by “continent” and “income level”, lowest prevalence of pooled survival was observed in Asia (six studies; pooled survival =36.0% with 95% CI = 19.01–52.15%; I^2^ = 97.4%; *p* < 0.001) and in low and middle income countries (six studies, pooled survival = 34.0% with 95% CI = 17.0–51.0%, I2 = 97.67%, *p* < 0.001) respectively.

**Conclusion:**

Although there was an extremely high heterogeneity among reported results (I^2^ = 96.7%), in this meta-analysis more than half of pediatric patients (54%) who underwent cardiopulmonary resuscitation for in-hospital cardiac arrest did not survived to hospital discharge. Therefore, developing further strategies and encouraging researches might be crucial.

**Supplementary Information:**

The online version contains supplementary material available at 10.1186/s13052-021-01058-9.

## Background

Cardiac arrest is a medical emergency characterized by cessation of cardiac activity. It leads to unresponsiveness; with no normal breathing, and no signs of circulation [1, 2]. It might be originated from the heart, lung, brain. Sometimes, toxic overdoses and severe infections might also be a cause [3, 4]. Cardiac arrest is a life-threatening condition and most probably ends up with death within a few minutes. In adults, cardiac arrest is commonly due to primary cardiac abnormalities. But, in children, it is most often the result of apnea or respiratory failure leading to bradycardia and pulseless electrical activity [2, 5]. Based on the location of arrest, it can be classified as in-hospital cardiac arrest—cessation of cardiac activity in hospitalized patients; or out-of-hospital cardiac arrest—an arrest occurred in outpatient settings (in the community) [6].

In-hospital cardiac arrest is a major public health issue which almost ends up with death both in adults and children [7]. The global prevalence of in-hospital cardiac arrest is not well documented. But, each year, nearly 1 million cases of cardiac arrest are recorded in Europe and United States [2, 8, 9]. Similarly, about 2–6% of children admitted to pediatric intensive care units are estimated to be victims of cardiac arrest [1]. About 60% of cardiac arrest cases among the pediatric population occurs in younger children or infants [4, 5]. Probability of survival after in-hospital cardiac arrest is low. More than 75% of the victims are estimated to die. One-third of the survivors will have neurological malfunctions [1, 5]. Hence, it is a threatening condition for families of the patient and healthcare providers. Furthermore, it is associated with devoting huge amounts of healthcare resources [7, 10].

Worldwide, different treatment strategies are implemented to reverse the ceased cardiac activity and to increase the probability of survival in children with cardiac arrest [4, 11, 12]. Cardiopulmonary resuscitation (CPR) is one of those strategies; comprising basic airway management, artificial ventilation, and chest compressions targeted to provide oxygen and nutrients for core organs: the heart, brain, and lungs. By doing so, CPR is expected to increase the probability of survival in patients with cardiac arrest [13]. It was implemented since 1966 with a well-defined, written procedural guidelines [14]. Additionally, prevention and prompt treatment of respiratory failure can prevent or reduce cardiac arrest in children [2].

Several studies were conducted to assess the success rate of CPR in terms of survival to hospital discharge among pediatric patients. However, reported results from those studies were inconsistent; with survival to hospital discharge ranging from 0 to 80% [15, 16]. Having a pooled prevalence of survival to hospital discharge after CPR is vital to develop further strategies targeted to increase probability of survival among patients with cardiac arrest. Despite of this, there is no current and updated pooled prevalence showing the overall survival to hospital discharge after CPR in pediatric patients [17]. Therefore, the purpose of this systematic review and meta-analysis was to assess the pooled prevalence of survival to hospital discharge among pediatric patients (age < 21 years) who underwent CPR for in-hospital cardiac arrest.

## Methods

### Reporting

This systematic review and meta-analysis has been presented according to the Preferred Reporting Items for Systematic Review and Meta-Analysis (PRISMA) guideline [18] (Additional file 1).

### Searching strategies

PubMed, Google Scholar, and Cochrane review databases were searched to identify relevant studies. The searching was also extended to repositories. Snowball searching was also employed to accommodate potentially related articles. The comprehensive searching strategy was developed by using different Boolean operators via Population Intervention Comparison and Outcome (PICO) standard questions. The core searching terms and phrases were “Cardiopulmonary Resuscitation”, “CPR”, “ECPR”, “In-hospital cardiac arrest”, “Success rate”, “Survival to hospital discharge” “Pediatric”, “Children”, “Infants” and “Neonates”. We had used these core terms in combination using the Boolean operators i.e. “AND” or “OR”. Filters were used to limit year of publication by applying the term “since 2016” or by bounding publication year range as “2016–2020.”

### Eligibility criteria

All published or unpublished observational studies (cross-sectional, case-control, and cohort designs) conducted to assess the prevalence of survival to hospital discharge after cardiopulmonary resuscitation in pediatric patients (age < 21 years) [19] were included. To have current evidence on survival to hospital discharge after CPR, only research articles published since 2016 were included. But, citations, research articles which did not report the outcome variable, if full text was not accessible, case presentations, or articles conducted on animals were excluded.

### Patient and public involvement

Patients or the public were not involved in the design, or conduct, or reporting, or dissemination plans of this meta-analysis.

### Outcome variable

The outcome variable was survival to hospital discharge in pediatric patients after CPR for in-hospital cardiac arrest.

### Quality assessment

After removing duplicate studies and screening potentially relevant articles, two independent authors (MB and AG) appraised the quality of eligible articles by using the nine score Newcastle-Ottawa Scale (NOS) for cohort studies [20] as a quality appraisal tool. Disagreements between appraisers were solved by taking their mean scores. Studies having “good” or “fair” quality based on the thresholds for converting the NOS score to the Agency for Healthcare Research and Quality (AHRQ) standards were considered as low risk and included in the final analysis; but studies with poor quality were excluded from the final analysis (Additional file 2).

### Data extraction and statistical analysis

The data was extracted and cleaned by using Microsoft Excel worksheet. After extraction, it was exported to STATA statistical software version 11.0. for further analysis; standard errors for the prevalence of the outcome variable (survival to hospital discharge) was calculated by using the binomial distribution formula [21]. The overall prevalence for the outcome variable was pooled based on the weighted inverse variance random-effects model at 95%CI. Results were presented using narrative synthesis, tables, and forest plots. Heterogeneity between included studies was assessed by the Inverse Variance (I^2^) with *p*-values. The values of I^2^, 25, 50, and 75% represent low, moderate, and high heterogeneity respectively [22]. Publication bias was assessed by funnel plots and Egger’s regression test [23]. Subgroup analysis by study area/ continent, income level and year of publication was also conducted.

## Results

### Study selection and characteristics of the included studies

**Fig. 1:**
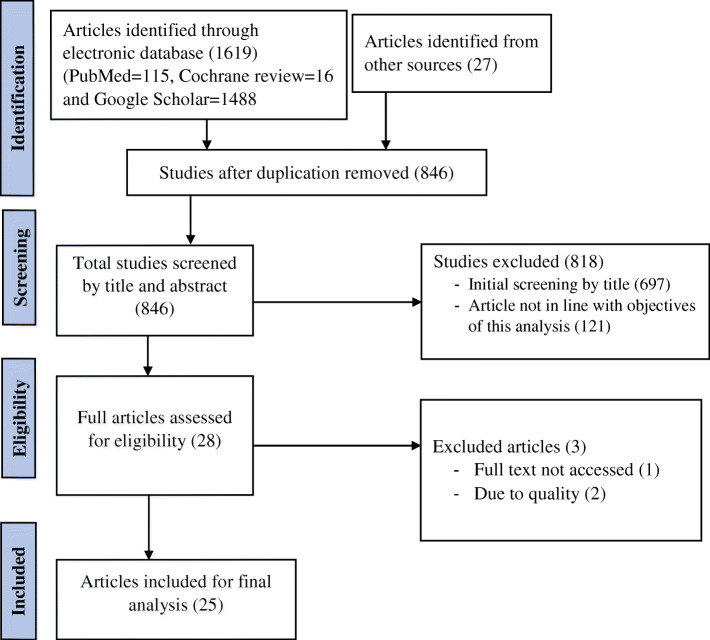
PRISMA flow diagram showing searching strategies, reasons for exclusion, and number of included research articles in this systematic review and meta-analysis, 2020

A total of 1646 research articles were retrieved from the comprehensive electronic searching. Of them, 25 articles [16, 24–47] were included in the final analysis (Fig. 1). All the included articles were cohort in their study design (Table 1).

**Table 1:** Characteristics of research articles included in this systematic review and meta-analysis, 2020

S. No	Author	Publi cation year	Country	Continent	Income level	Sample size	SHD (%)
1	Appiah J et al. [24]	2018	South Africa	Africa	Upper-middle income	83	62.65
2	Shikuku DN et al. [25]	2018	Kenya	Africa	Lower-middle income	24	45.83
3	Alten et al. [26]	2017	North America	America	High income	470	54.04
4	Anton-Martin P et al. [27]	2020	United states	America	High income	73	43.84
5	Barbaro RP et al. [28]	2017	United states	America	High income	8575	41.94
6	Berg et al. [29]	2016	United states	America	High income	139	45.32
7	Berg et al. [30]	2018	United states	America	High income	164	46.95
8	Beshish AG et al. [31]	2018	Michigan	America	High income	80	47.50
9	Brown S et al. [32]	2018	Washington	America	High income	52	48.08
10	Burke CR et al. [33]	2017	United states	America	High income	53	49.06
11	Foglia et al. [34]	2017	Pennsylvania	America	High income	113	61.06
12	Geisser D et al. [35]	2019	Massachusetts	America	High income	295	41.69
13	Holmberg et al. [36]	2019	United states	America	High income	13,184	45.50
14	Hornik et al. [37]	2016	North America	America	High income	2231	50.52
15	Shakoor A et al. [38]	2019	New York	America	High income	70	54.29
16	Torres-Andres et al. [39]	2018	United states	America	High income	55	67.27
17	Assar S et al. [40]	2016	Iran	Asia	Upper-middle income	279	11.83
18	Chen GL et al. [41]	2018	Asia pacific	Asia	Low income	321	50.78
19	Erek et al. [42]	2017	Turkey	Asia	Upper-middle income	25	20.00
20	Kabbani et al. [16]	2019	Saudi Arabia	Asia	High income	15	80.00
21	Mok YH et al. [43]	2016	Singapore	Asia	High income	51	45.10
22	Rathore V et al. [44]	2016	North India	Asia	Lower-middle income	314	14.01
23	Adamski J et al. [45]	2016	Poland	Europe	High income	285	53.33
24	Kramer P et al. [46]	2020	Germany	Europe	High income	72	36.11
25	Skellett S et al. [47]	2020	United kingdom	Europe	High income	1456	54.19

*SHD* Survival to Hospital Discharge

### Survival to hospital discharge

**Fig. 2:**
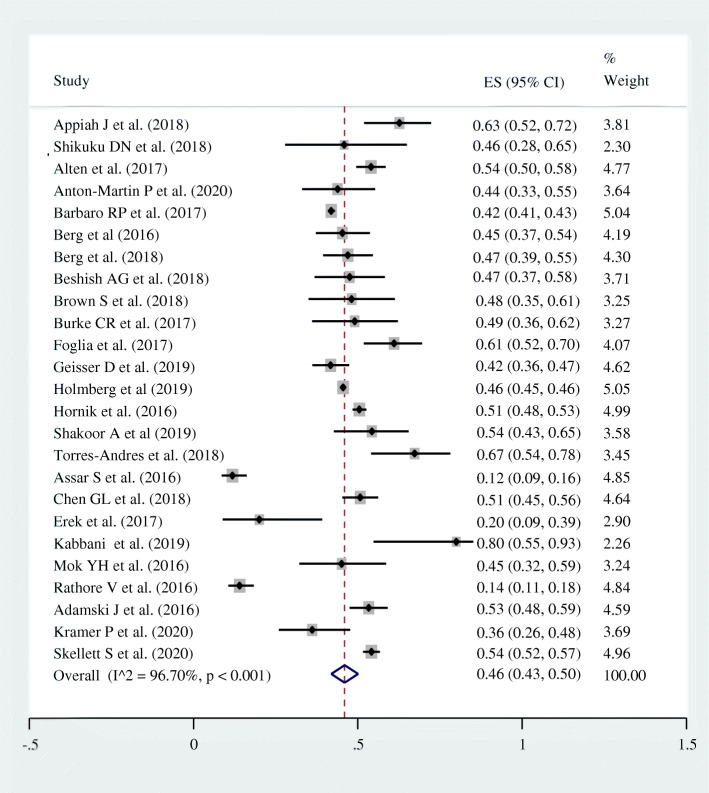
Forest plot showing the pooled prevalence of survival to hospital discharge among pediatric patients who underwent cardiopulmonary resuscitation for in-hospital cardiac arrest, 2020

Twenty-five studies with a total sample size of (*n* = 28,479) had reported the prevalence of survival to hospital discharge in pediatric patients after CPR for in-hospital cardiac arrest with a lowest and highest prevalence of 11.8 and 80% respectively. The pooled prevalence of survival to hospital discharge in those patients was found to be 46.0% (95% CI = 43.0–50.0%; I^2^ = 96.7%; *p* < 0.001) (Fig. 2).

### Heterogeneity

The inverse variance (I^2^) was 96.7% with a *p*-value of < 0.001 (Fig. 2); suggesting the presence of heterogeneity on the reported prevalence of survival to hospital discharge in pediatric patients who underwent CPR for in-hospital cardiac arrest among included studies.

### Sensitivity analysis

By using the random-effects model, a leave-one-out sensitivity analysis was conducted to examine if the pooled magnitude of survival to hospital discharge was greatly impacted by the result of a single study. But, all the results of this sensitivity analysis were within the 95% CI limits of the pooled magnitude (41.9–50.1%); suggesting that there was no influential study potentially affected the observed pooled magnitude of survival to hospital discharge (Additional file 3).

### Publication bias

Since the funnel plot showed symmetrical distribution (Additional file 4), and Egger’s regression test was found insignificant (0.75), there was no evidence for publication bias in the included studies.

### Subgroup analysis

**Fig. 3:**
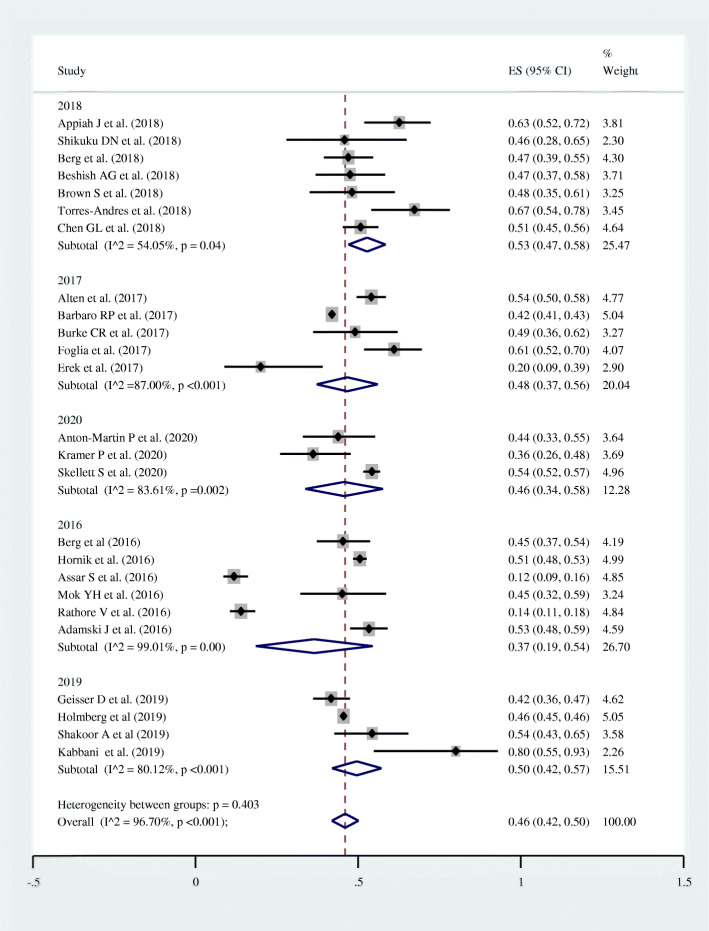
Forest plot showing the pooled survival to hospital discharge by publication year among pediatric patients who underwent cardiopulmonary resuscitation for in-hospital cardiac arrest, 2020

Subgroup analysis was conducted by “year of publication” and the peak prevalence of survival was observed among studies published in 2018 with a pooled prevalence of 53.0%. The pooled prevalence of survival to hospital discharge was 37.0, 48.0, 50.0, and 46.0% among studies published in 2016, 2017, 2019, and 2020 respectively (Fig. 3).

**Fig. 4:**
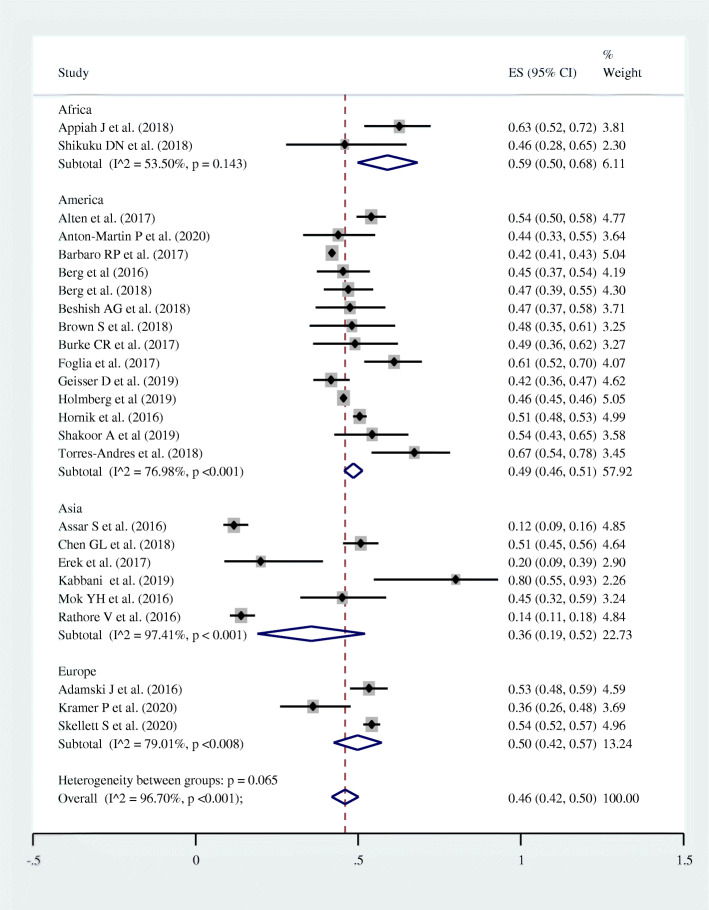
Forest plot showing the intercontinental disparities in terms of survival to hospital discharge among pediatric patients who underwent cardiopulmonary resuscitation for in-hospital cardiac arrest, 2020

Subgroup analysis was also conducted by “study area/ continents” to observe intercontinental disparities on survival to hospital discharge in pediatric patients who underwent CPR for in-hospital cardiac arrest. Lowest magnitude of pooled survival was observed in Asia (six studies; pooled survival =36.0% with 95% CI = 19.0–52.0%; I^2^ = 97.4%; *p* < 0.001) (Fig. 4).

**Fig. 5:**
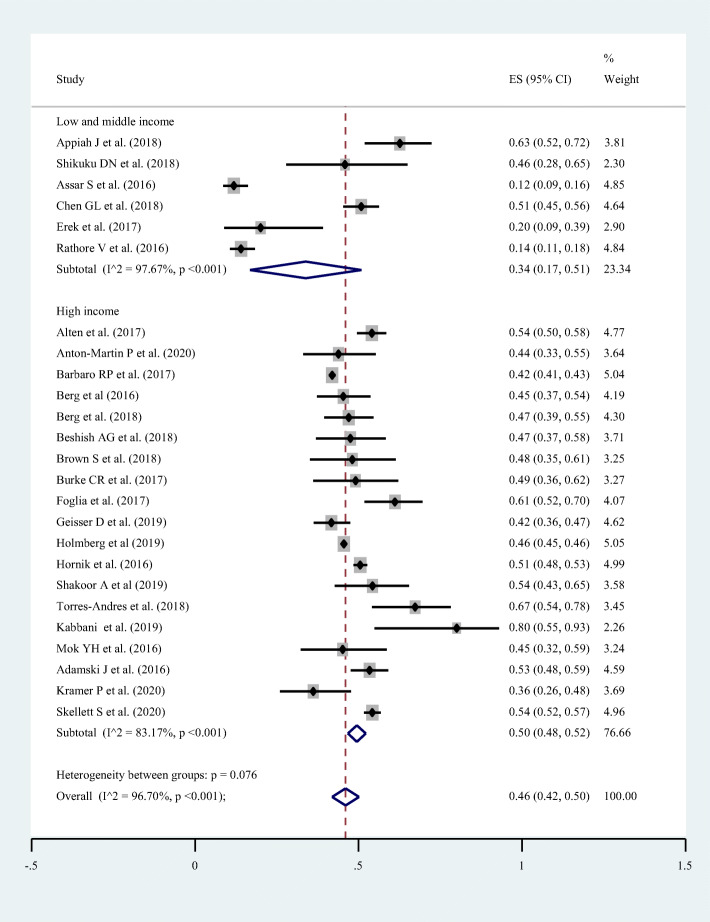
Forest plot showing the disparities in terms of survival to hospital discharge among pediatric patients who underwent cardiopulmonary resuscitation in low and middle income and high income countries, 2020

Subgroup analysis was also conducted by “income level” to observe disparities on survival among low and middle income countries and high income countries. Lowest prevalence of pooled survival was observed in Low and middle income countries (six studies; pooled survival =34.0% with 95% CI = 17.0–51.0%; I^2^ = 97.7%; *p* < 0.001) (Fig. 5).

## Discussion

A total of twenty-five research articles were included in this systematic review and meta-analysis. Nineteen studies were from high income countries and the remaining six studies were from low and middle income countries (Table 1). This indicates that studies addressing the issue of survival to hospital discharge in pediatric patients who underwent CPR for in-hospital cardiac arrest were limited in low and middle income countries. Therefore, the authors of this meta-analysis believe in a need for further research from those countries.

In this meta-analysis, the pooled prevalence of survival to hospital discharge in pediatric patients who underwent CPR for in-hospital cardiac arrest was found to be 46.0% (95% CI = 42.0–50.0%; I^2^ = 96.8%; *p* < 0.001). This was in line with studies conducted in Spain [48] and United States [49]; as they had reported post-CPR survival to hospital discharge in children to be 41 and 43.4% respectively. Lower prevalence of post-CPR survival to hospital discharge in children were reported from studies conducted in Taiwan (20.9%) [50] and China (28.2%) [51]. Findings of this meta-analysis was also higher than a global meta-analysis conducted by Phillips RS et al. [52]; as it had reported the pooled prevalence of survival to hospital discharge after CPR in children to be 37.2%. Those inconsistencies might be due to differences in the study period.

Extremely high heterogeneity (I^2^ = 96.7%) was observed among included studies in this meta-analysis. Wider age ranges (birth - 21 years) among included studies might have been a possible source for this heterogeneity. Differences in CPR-type might have been also a possible source of heterogeneity; as eight studies used advanced technologies (Extracorporeal cardiopulmonary resuscitation (ECPR)) and others used conventional CPR or the combination of the two. Differences in etiology of the cardiac arrest might be also another possible source of this extreme heterogeneity. Whereas two studies included patients after surgery, some studies were conducted on patients with unspecified etiology of arrest, and other studies were conducted on neonates after birth. Differences in unit of the health institution where CPR was performed might have been also a possible source; as studies from pediatric intensive care units, neonatal intensive care units, cardiac intensive care units, general wards, and emergency departments were included.

Another possible source for the observed heterogeneity might be differences in time lag between conventional CPR and extracorporeal life support (ECLS), as one study reported a 30-min lag, one observed a 10-min lag, while others had not specified. Differences in inclusion criteria applied in individual included studies might be also a possible source for the observed heterogeneity; as some studies included patients who had CPR for at least 10 min, while some included patients who had CPR for at least 1 min. Differences in socioeconomic status of the study area and time period might have been also a source for this heterogeneity. To reduce the higher heterogeneity observed in this meta-analysis, authors had employed subgroup analysis based on the possible sources of heterogeneity discussed above. But, unfortunately subgroup analysis was impossible due to overlaps in category of variables or missing information, except for publication year, continent/ study area, and income level.

Based on the subgroup analysis conducted by year of publication, the lowest prevalence of post-CPR survival (37.0%) was recorded among articles published in 2016. On the other hand, the highest prevalence of survival was recorded among studies published in 2018. The general survival had increased from 36.5% in 2016 to 45.7% in 2020; due to the improvement in post CPR survival. This improvement might be due to technological advancements with time. In line with this meta-analysis, literature had also indicated that survival to hospital discharge after CPR had increased in the last few decades [49, 53–56].

Intercontinental disparities in survival to hospital discharge were observed in this meta-analysis. The lowest post-CPR survival (36.0%) was recorded in Asia; which was consistent with a meta-analysis conducted by Yan et al. [57]. This might be due to lower socioeconomic status in Asia; as four of the six included studies from Asia were from low and middle income countries. On the other hand, the highest prevalence of survival was observed in Africa (59.0%); but it was based on two studies with a total sample size of 107 which makes the observed pooled prevalence of survival to be a little evidence, and to be regarded with suspicion. Post-CPR survival to hospital discharge was nearly similar in America (49.0%) and Europe (50.0%). This might be due to similarities in socioeconomic status; as all included studies were from high income countries both in studies from America and Europe.

Based on the subgroup analysis by “income level”, lower magnitude of post-CPR survival (34.0%) was observed in low and middle income countries. In fact, low and middle income countries cannot afford prices for advanced CPR technologies like extracorporeal membrane oxygenation (ECMO) and cardioversion as can high income countries. Therefore, as revealed by this meta-analysis, low and middle income countries will have lower post-CPR survival than high income countries. Literature had also showed survival after CPR to be higher in developed countries than developing countries [57–59] which might be attributed to differences in technological and medical advancement. Additionally, a late referral to hospital for respiratory problems or failure could be the explanation of lowest survival in low and middle income countries.

### Strength and limitations

In this meta-analysis, authors had used internationally qualified tools for evaluating the quality of included studies. Authors had also employed test for publication bias and subgroup analysis. However, this meta-analysis might not be free of limitations: specifically, publication bias might have occurred.

### Conclusion and future implications

More than half (54%) of CPR procedures in pediatric patients who underwent CPR for in-hospital cardiac arrest are unsuccessful in terms of survival to hospital discharge. Therefore, devoting efforts to develop further strategies based on new evidence might be crucial to improve clinical outcomes of CPR in children. Additionally, prevention and prompt treatment of respiratory problems should be emphasized to cardiac arrest in children. Researches from low and middle income countries addressing the issue of post-CPR survival were limited. Hence, low and middle income countries should develop CPR-registry systems like high income countries and encourage research on this issue. Furthermore, factors associated with survival to hospital discharge after CPR were not addressed in this meta-analysis. As a result, authors of this meta-analysis recommend to investigate further on this issue.

## Supplementary Information

**Additional file 1.** PRISMA Checklist

**Additional file 2: Table S1.** The Newcastle-Ottawa Scale based quality assessment of included studies, 2020

**Additional file 3: Table S2.** A leave-one-out sensitivity analysis among included studies showing if the pooled magnitude of survival to hospital discharge was greatly impacted by the result of a single study, 2020

**Additional file 4.** Funnel plot showing the absence of publication bias (the symmetrical distribution of included research articles by the prevalence of survival to hospital discharge), 2020

## Data Availability

The datasets used and/or analyzed during the current study are available within the manuscript.
