# Supplementary material for: Survival of pediatric patients after cardiopulmonary resuscitation for in-hospital cardiac arrest: a systematic review and meta-analysis
Source: Ital J Pediatr. 2021 May 29;47:118. doi: 10.1186/s13052-021-01058-9 (PMC8164331; doi:10.1186/s13052-021-01058-9)
Supplement: Supplementary file 2 — Additional file 2: Table S1. The Newcastle-Ottawa Scale based quality assessment of included studies, 2020 [file 13052_2021_1058_MOESM2_ESM.pdf]

Table S1: The Newcastle-Ottawa Scale based quality assessment of included studies, 2020

S. No.	Authors' name	Publi-cation year	Study area	Study design	Domain			Total	Quality
					Selection	Compara-bility	Outcome		
1	Appiah J et al. (24)	2018	Africa	Cohort	2/4	1/2	2/3	6/9	Fair
2	Shikuku DN et al. (25)	2018	Africa	Cohort	3/4	1/2	2/3	7/9	Good
3	Alten et al. (26)	2017	America	Cohort	3/4	1/2	2/3	8/9	Good
4	Anton-Martin P et al. (27)	2020	America	Cohort	3/4	1/2	2/3	7/9	Good
5	Barbaro RP et al. (28)	2017	America	Cohort	3/4	1/2	2/3	7/9	Good
6	Berg et al. (29)	2016	America	Cohort	3/4	1/2	2/3	7/9	Good
7	Berg et al. (30)	2018	America	Cohort	3/4	1/2	2/3	8/9	Good
8	Beshish AG et al. (31)	2018	America	Cohort	3/4	1/2	2/3	7/9	Good
9	Brown S et al. (32)	2018	America	Cohort	3/4	1/2	2/3	7/9	Good
10	Burke CR et al. (33)	2017	America	Cohort	3/4	1/2	2/3	6/9	Fair
11	Foglia et al. (34)	2017	America	Cohort	3/4	1/2	2/3	7/9	Good
12	Geisser D et al. (35)	2019	America	Cohort	3/4	1/2	2/3	7/9	Good
13	Holmberg et al. (36)	2019	America	Cohort	3/4	1/2	2/3	8/9	Good
14	Hornik et al. (37)	2016	America	Cohort	3/4	1/2	2/3	7/9	Good
15	Shakoor A et al. (38)	2019	America	Cohort	3/4	1/2	2/3	6/9	Fair
16	Torres-Andres et al. (39)	2018	America	Cohort	3/4	1/2	2/3	7/9	Good
17	Assar S et al. (40)	2016	Asia	Cohort	3/4	1/2	2/3	8/9	Good
18	Chen GL et al. (41)	2018	Asia	Cohort	3/4	1/2	2/3	7/9	Good
19	Erek et al. (42)	2017	Asia	Cohort	3/4	1/2	2/3	6/9	Fair
20	Kabbani et al. (16)	2019	Asia	Cohort	3/4	1/2	2/3	7/9	Good
21	Mok YH et al. (43)	2016	Asia	Cohort	3/4	1/2	2/3	6/9	Fair
22	Rathore V et al. (44)	2016	Asia	Cohort	3/4	1/2	2/3	8/9	Good
23	Adamski J et al. (45)	2016	Europe	Cohort	3/4	1/2	2/3	8/9	Good
24	Kramer P et al. (46)	2020	Europe	Cohort	3/4	1/2	2/3	7/9	Good
25	Skellett S et al. (47)	2020	Europe	Cohort	3/4	1/2	2/3	7/9	Good
	***Thresholds for converting the Newcastle-Ottawa scales score to the Agency for Healthcare Research and Quality (AHRQ) standards (as good, fair, and poor): - Good quality**: 3 or 4 stars in selection domain AND 1 or 2 stars in comparability domain AND 2 or 3 stars in outcome/exposure domain; **Fair quality**: 2 stars in selection domain AND 1 or 2 stars in comparability domain AND 2 or 3 stars in outcome/exposure domain; **Poor quality**: 0 or 1 star in selection domain OR 0 stars in comparability domain OR 0 or 1 stars in outcome/exposure domain*								
