# Supplementary material for: Survival of pediatric patients after cardiopulmonary resuscitation for in-hospital cardiac arrest: a systematic review and meta-analysis
Source: Ital J Pediatr. 2021 May 29;47:118. doi: 10.1186/s13052-021-01058-9 (PMC8164331; doi:10.1186/s13052-021-01058-9)
Supplement: Supplementary file 3 — Additional file 3: Table S2. A leave-one-out sensitivity analysis among included studies showing if the pooled magnitude of survival to hospital discharge was greatly impacted by the result of a single study, 2020 [file 13052_2021_1058_MOESM3_ESM.pdf]

Table S2: A leave-one-out sensitivity analysis among included studies showing if the pooled magnitude of survival to hospital discharge was greatly impacted by the result of a single study, 2020

Study omitted	Estimate	95% CI
Appiah J et al. (24)	0.45	0.41, 0.49
Shikuku DN et al. (25)	0.46	0.42, 0.50
Alten et al. (26)	0.46	0.41, 0.50
Anton-Martin P et al. (27)	0.46	0.42, 0.50
Barbaro RP et al. (28)	0.47	0.41, 0.52
Berg et al. (29)	0.46	0.42, 0.50
Berg et al. (30)	0.46	0.42, 0.50
Beshish AG et al. (31)	0.46	0.42, 0.50
Brown S et al. (32)	0.46	0.42, 0.50
Burke CR et al. (33)	0.46	0.42, 0.50
Foglia et al. (34)	0.45	0.41, 0.49
Geisser D et al. (35)	0.46	0.42, 0.50
Holmberg et al. (36)	0.46	0.41, 0.52
Hornik et al. (37)	0.46	0.41, 0.50
Shakoor A et al. (38)	0.46	0.42, 0.50
Torres-Andres et al. (39)	0.45	0.41, 0.49
Assar S et al. (40)	0.48	0.44, 0.51
Chen GL et al. (41)	0.46	0.42, 0.50
Erek et al. (42)	0.47	0.43, 0.51
Kabbani et al. (16)	0.45	0.41, 0.49
Mok YH et al. (43)	0.46	0.42, 0.50
Rathore V et al. (44)	0.47	0.44, 0.51
Adamski J et al. (45)	0.46	0.41, 0.50
Kramer P et al. (46)	0.46	0.42, 0.51
Skellett S et al. (47)	0.46	0.41, 0.50
Overall	0.46	0.43, 0.50
Abbreviations: CI; Confidence Interval		
