# Supplementary figures and images for: Survival of pediatric patients after cardiopulmonary resuscitation for in-hospital cardiac arrest: a systematic review and meta-analysis
Source: Ital J Pediatr. 2021 May 29;47:118. doi: 10.1186/s13052-021-01058-9 (PMC8164331; doi:10.1186/s13052-021-01058-9)

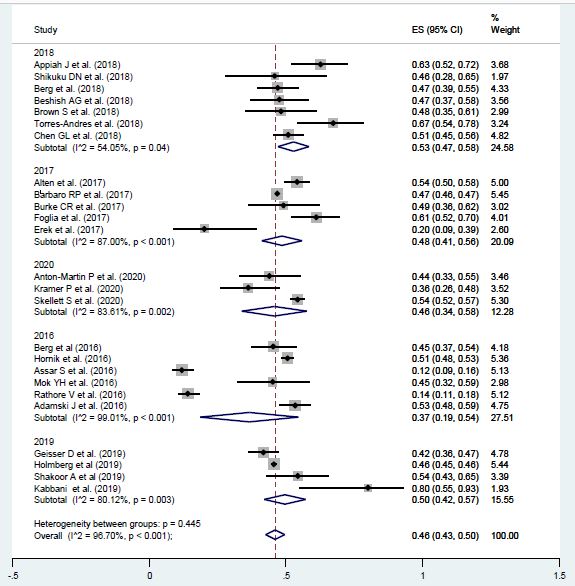

Supplement: Supplementary file 4 — Additional file 4. Funnel plot showing the absence of publication bias (the symmetrical distribution of included research articles by the prevalence of survival to hospital discharge), 2020 [file 13052_2021_1058_MOESM4_ESM.jpg]
